# Nutrient depletion and TOR inhibition induce 18S and 25S ribosomal RNAs resistant to a 5′-phosphate-dependent exonuclease in *Candida albicans* and other yeasts

**DOI:** 10.1186/s12867-018-0102-y

**Published:** 2018-01-19

**Authors:** Jacob Fleischmann, Miguel A. Rocha

**Affiliations:** 1 Department of Medicine, Greater Los Angeles VA Healthcare System, Los Angeles, CA USA; 2 Research Division, Greater Los Angeles VA Healthcare System, Los Angeles, CA USA; 3 Department of Medicine, David Geffen School of Medicine at UCLA, Los Angeles, CA USA; 4 16111 Plummer St, North Hills, CA 91343 USA

**Keywords:** RNA, Ribosomal, *Candida albicans*, Terminator, Exonuclease

## Abstract

**Background:**

Messenger RNA (mRNA) represents a small percentage of RNAs in a cell, with ribosomal RNA (rRNA) making up the bulk of it. To isolate mRNA from eukaryotes, typically poly-A selection is carried out. Recently, a 5´-phosphate-dependent, 5´→3´ processive exonuclease called Terminator has become available. It will digest only RNA that has a 5´-monophosphate end and therefore it is very useful to eliminate most of rRNAs in cell.

**Results:**

We have found that in the pathogenic yeast *Candida albicans*, while 18S and 25S components isolated from yeast in robust growth phase are easily eliminated by Terminator, those isolated from cells in the nutritionally diminished stationary phase, become resistant to digestion by this enzyme. Additional digestions with alkaline phosphatase, tobacco pyrophosphatase combined with Terminator point toward the 5′-prime end of 18S and 25S as the source of this resistance. Inhibition of TOR by rapamycin also induces resistance by these molecules. We also find that these molecules are incorporated into the ribosome and are not just produced incidentally. Finally, we show that three other yeasts show the same behavior.

**Conclusions:**

Digestion of RNA by Terminator has revealed 18S and 25S rRNA molecules different from the accepted processed ones seen in ribosome generation. The reason for these molecules and the underlying mechanism for their formation is unknown. The preservation of this behavior across these yeasts suggests a useful biological role for it, worthy of further inquiry.

## Background

Ribosomes, the protein producing organelles of eukaryotic cells, are composed of four individual ribosomal RNAs (rRNA) and combined with 79 proteins [1]. In yeast, including in *Candida albicans* the genes coding for rRNA (rDNA) are repeated multiple times in a tandem fashion [2]. The ultimate construction of this organelle is a result of the combined efforts of three RNA polymerases [3]. RNA polymerase I (Pol I) copies full length polycistronic transcripts off the ribosomal RNA gene (rDNA) [4]. These transcripts get immediately processed into large (25S) and small (18S, 5.8S) subunits as transcribed internal spacers (ITS1, ITS2) and external spacers (5′-ETS, 3′-ETS) are removed by a combination of endonucleolytic and exonucleolytic cleavages [5]. RNA polymerase III (Pol III) copies a small (5S) subunit off a sequence located between tandem repeats from the opposite strand [6]. Finally, RNA polymerase II copies the messenger RNAs (mRNA) from their respective genes and their translated products make up the protein component of the ribosome [7]. All three RNA polymerases are regulated by TOR kinase, adjusting ribosome production to match the metabolic needs of the cell [8].

Studies of ribosomal RNA (rRNA) in *Candida albicans* have been a focus in our laboratory [9–11]. We recently obtained Terminator (Epicentre/Illumina Co.), a 5´-phosphate-dependent processive 5´→3´ exonuclease that digests only RNA that has a 5´-monophosphate end [12]. It does not digest RNA that has a 5´-triphosphate, 5´-cap or 5´-hydroxyl group at its 5′-side. Its primary use is to isolate mRNA by digesting away 18S and 25S rRNAs, thus avoiding the need for poly-A selection (http://www.epibio.com/enzymes/nucleases-glycosylases-dna-binding-proteins/rna-exonucleases/terminator-5-phosphate-dependent-exonuclease) [13]. Surprisingly, we noticed variability in the enzyme’s behavior and decided to look at it more carefully. We report here that this enzyme is highly efficient in digesting 18S and 25S rRNAs isolated from yeast in mid-log growth phase. However, as organisms shift to a stationary phase, some of 18S and 25S rRNAs become resistant to Terminator cleavage. We show that the resistant rRNAs are incorporated into the ribosome. Interestingly, the same resistance develops when TOR activity is inhibited by rapamycin. Finally, we observed a similar pattern in other yeasts, which points toward a possible biological role for the development of this resistance.

## Results

**Fig. 1:**
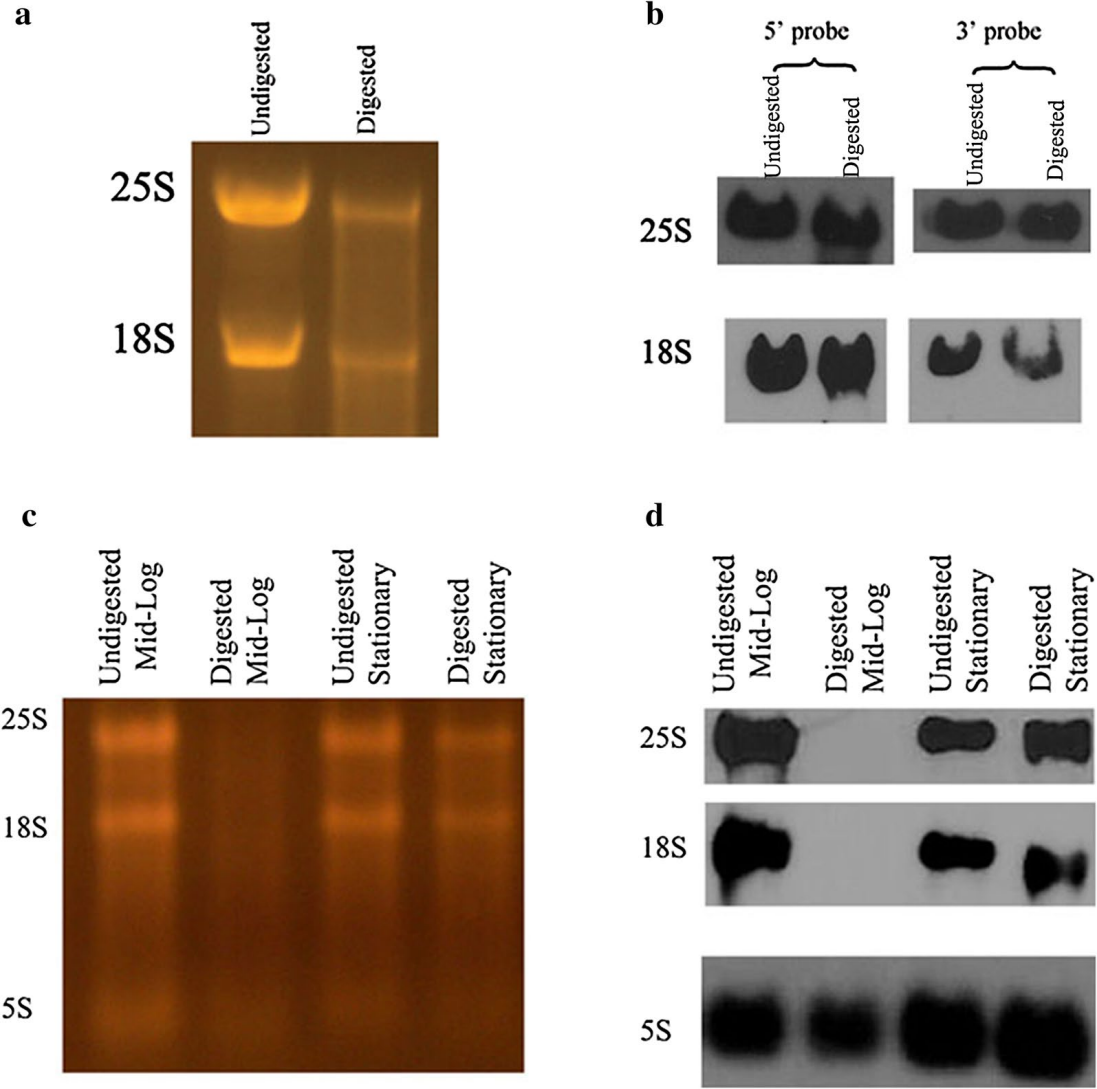
Total RNA from yeast in stationary and mid log growth phase treated or untreated with Terminator. **a** SYBR-Gold stained gel showing RNA treated with Terminator along with untreated RNA. Significant portions of 25S and 18S rRNA are protected from digestion. All conditions started out with the same amount of RNA. **b** Northern blot, using probes specific for either 5′ or 3′ ends of 25S and 18S ribosomal subunits confirming them to be the large and small subunits of rRNA protected from Terminator digestion. **c** SYBR-Gold stained gel displaying RNA isolated at mid log and stationary phases and treated with or without Terminator. **d** Northern Blot using 25S, 18S and 5S probes. Ribosomal 5S was used as loading control

Our initial use of Terminator was with total RNA isolated from *Candida albicans* SC5314 grown overnight (16 h), therefore in the stationary phase, and we found that both 18S and 25S resisted the exonuclease digestion. Figure 1a depicts an example of this phenomenon where equivalent amounts of RNA digested with Terminator or undigested were electrophoresed through a formaldehyde/agarose gel and SYBR-Gold stained. 18S and 25S bands are seen in the two undigested and digested RNA lanes. To insure full enzyme efficiency, we did digestions with up to three times the enzyme to substrate ratios recommended, and over longer time periods, with these bands maintaining their presence (data not shown). Northern blot analysis confirmed that the rRNAs protected from the exonuclease treatment were indeed 18S and 25S (Fig. 1b). Furthermore, our Northern analysis, utilizing probes specific for 5′ and 3′ ends for both 18S and 25S combined with the size of resistant molecules being the same as digestible molecules, indicated no size change during resistance development (see Table 1 for probe sequences). To see if growth phase would make a difference, we looked at cells grown to mid-log phase (incubated in fresh YPD for 4–6 h). As can be seen in Fig. 1c, Terminator fully eliminated all rRNA molecules from RNA obtained from cells in mid-log phase. The number of yeast cells from which RNA was obtained was the same for both time points. Northern blotting (Fig. 1d) confirmed these observations. Furthermore, a probe specific for 5S rRNA, a product of RNA polymerase III (Pol III), confirmed that our observed differences were not related to differences in amounts of RNA loaded. The data assured us that these observed molecules were truly resistant to Terminator.

**Table 1:** Probe sequences used for Northern blotting

Name and sequence	Chromosomal coordinates^a^
18S 5′ probe	
GCCAGTAGTCATATGCTTGTCTCAAAGATTAAGCCATGCATGTCTAAGTATAAGCAATTTATACAGTGAAACTGCGAATGGCTCATTAAATCAGTTATCGTTTATTTGATAGTACCTTACTACTTGGATAACCGTGGTAATTCTAGAGCTAATACATGCT	1891221–1891411
18S 3′ probe	
TCCGGATTGGTTTAGGAAAGGGGGCAACCTCATTCTGGAACCGAGAAGCTGGTCAAACTTGGTCATTTAGAGGAAGTAAAAGTCGTAACAAGGTTTCCGTAGGTGAACCTGCGGAAGGATCATTA	1892897–1893017
25S 5′ probe	
ATCAGGTAGGACTACCCGCTGAACTTAAGCATATCAATAAGCGGAGGAAAAGAAACCAACAGGGATTGCCTCAGTAGCGGCGAGTGAAGCGGCAAAAGCTCAAATTTGAAATCTGGCGTCTTTGGCGTCCGAGTTGTAATTTGAAGAAGGTATCTTTGGGCCCGGCTCTTGTCTATGTTCCTTGGAACAGGACGTCACAGAGGGTGAGAATCCCGTGCGATGAGATGAC	1893459–1893685
25S 3′ probe	
AACATGCGCGGGGATAAATCCTTTGCATACGACTTAGATGTACAACGGAGTATTGTAAGCAGTAGAGTAGCCTTGTTGTTACGATCTGCTGAGATTAAGCTCTTGTTGTCTGATTTG	1896713–1896827
5S probe	
GGTTGCGGCCATATCTAGCAGAAAGCACCGTTCCCCGTTCGATCAACCGTAGTTAAGCTGCTAAGAGCAATACCGAGTAGTGTAGTGGGAGACCATACGCGAAACTATTGTGCTGCAATCT	1889054–1888934
ITS-2 probe	
TCGTTTCTCCCTCAAACCGCTGGGTTTGGTGTTGAGCAATACGACTTGGGTTTGCTTGAAAGACGGTAGTGGTAAGGCGGGATCGCTTTGACAATGGCTTAGGTCTAACCAAAAACATTGCTTGCGGCGGTAACGTCCACCACGTATATCTTCAAACTTTG	1893319–1893469

^a^ http://www.candidagenome.org/

**Fig. 2:**
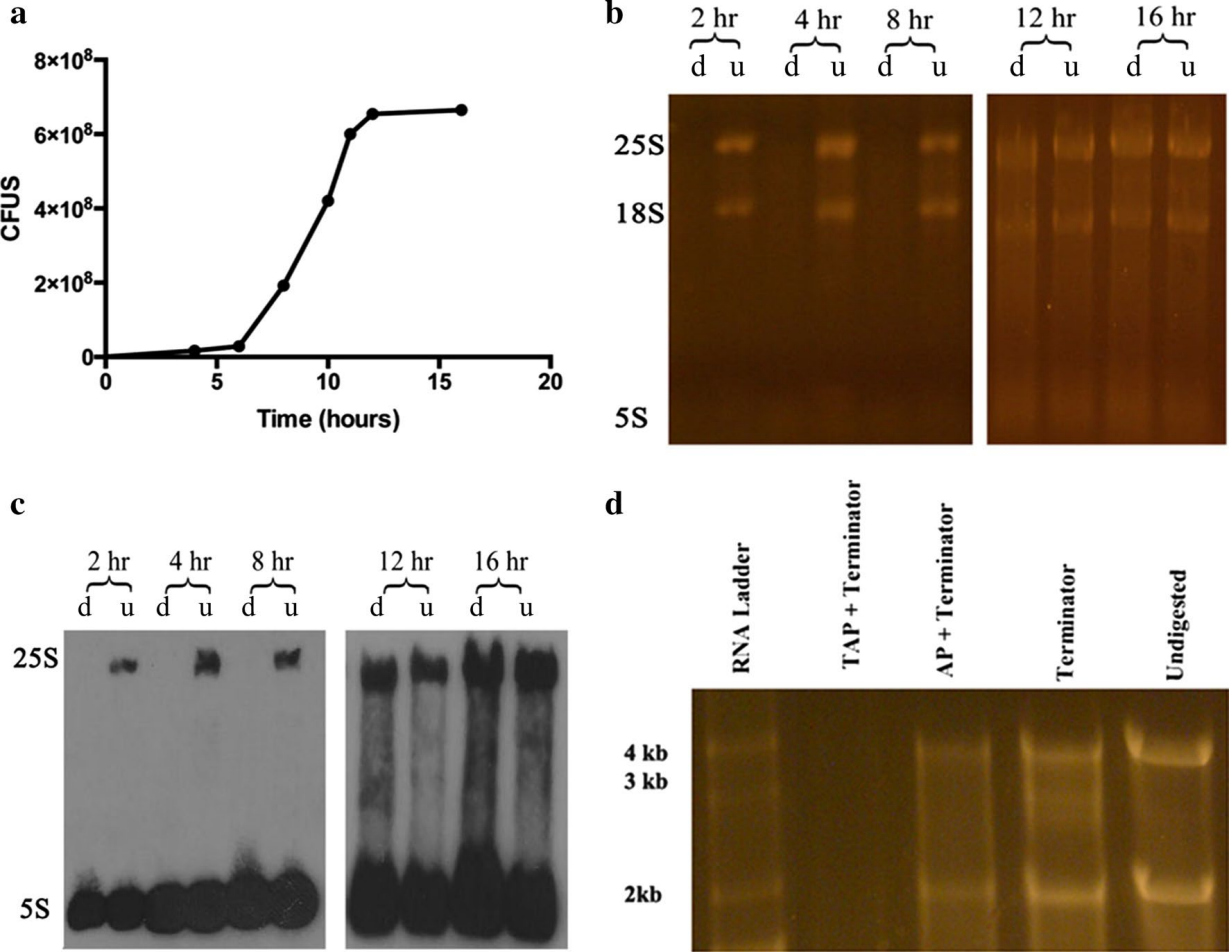
18S and 25S rRNAs protected from Terminator digestion as organisms shift to stationary phase and analysis of 5′-end for possible role in protection. **a** *C. albicans* growth curve at 30 °C in YPD medium. **b** SYBR gold stained gel of total RNA isolated at different time points either digested with Terminator (d) or undigested (u). **c** Northern blot of gel seen in **a** using 25S and 5S specific probes (Table 1). **d** RNA digested with Terminator alone or following TAP or AP digestion. As can be seen both 25S and 18S remain protected after AP digestion but get eliminated after TAP digestion. *CFUS* colony forming units

We did a more detailed timing experiment to see how the resistance development was related to growth phase (Fig. 2a). As can be seen in Fig. 2b, Terminator eliminated both 18S and 25S bands while the organism was in the mid-log phase. The protected molecules made their appearance as the organism was shifting from mid-log to stationary growth. The intensity of the preserved bands increased with incubation time and appeared to be close to the undigested one by 16 h. This is interesting since Pol I transcription and processing of rRNA are usually downregulated with decreasing nutrition availability. Northern blotting with a 25S specific probe again confirmed that this rRNA was indeed 25S (Fig. 2c).

We carried out serial enzyme digestion experiments to examine whether the resistance to Terminator resided at the 5′-position of the rRNAs. In one experiment, we used tobacco acid pyrophosphatase (TAP) which cuts specifically between the α and β phosphate groups resulting in a monophosphate 5′ end. When TAP was followed by Terminator treatment, the previously preserved bands were eliminated (Fig. 2d). Another serial enzyme digestion included alkaline phosphatase (AP) followed by Terminator; in this case the rRNAs remained protected (Fig. 2d). This could have been either due to further modification in rRNA protecting against both AP and Terminator, or more than one phosphate being digested by AP (https://www.neb.com/products/m0290-alkaline-phosphatase-calf-intestinal-cip) resulting in 5′-OH, again preventing exonuclease digestion.

**Fig. 3:**
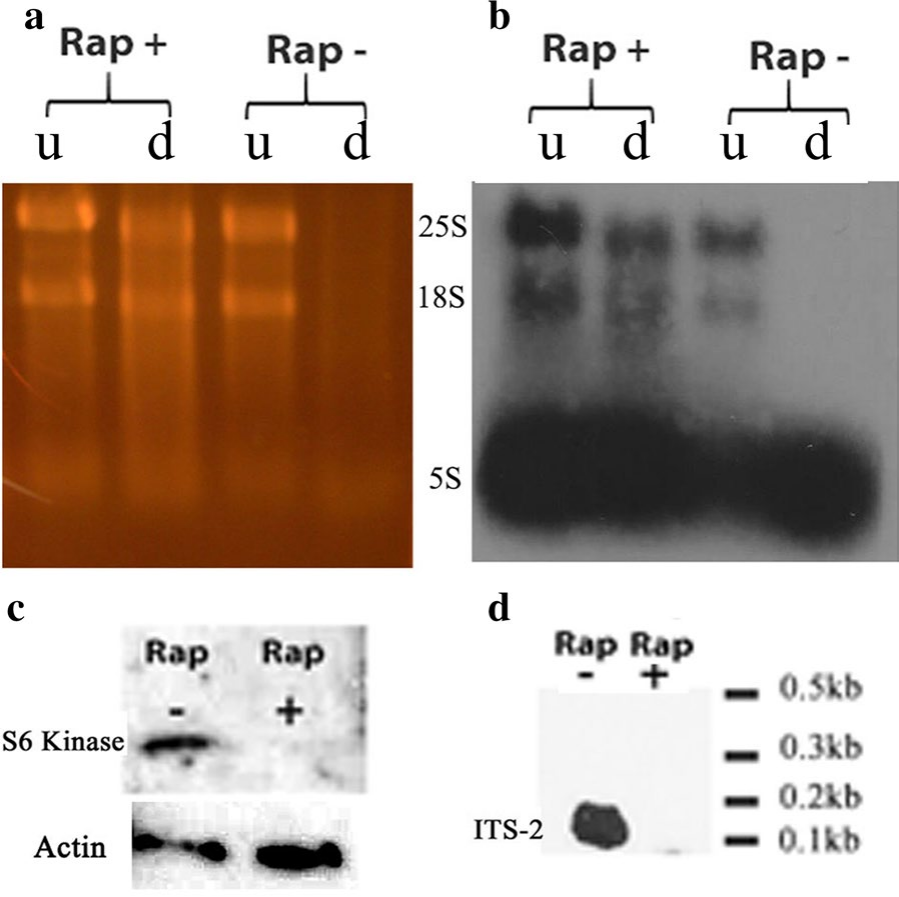
TOR inhibition also leads to Terminator resistance. **a** SYBR gold stained gel showing RNA from cells grown in fresh YPD with rapamycin at 1 µg/ml for 6 h at 30 °C. Controls (Rap-) were cells in fresh YPD without rapamycin for 6 h at 30 °C. RNA was either undigested (u) or digested by Terminator (d). **b** Northern blot from **a**. **c** Western blot performed on protein isolated and probed with S6 kinase and actin specific antibodies from cells grown as in **a** with and without rapamycin treatment. **d** Northern blot performed with ITS2 specific probe using RNA from cells grown under the same conditions as in **a** (see Table 1 for probe sequences). All blots started with the same amount of total RNA or protein loaded for all conditions

To see whether the transcription of these molecules was related to TOR activity, we exposed cells to rapamycin (1 µg/ml) for the same length of time as control cells (6 h), both in fresh YPD. Total RNA was extracted from the two conditions and submitted to Terminator. As can be seen on Fig. 3a, rapamycin exposed organisms yielded 18S and 25S molecules resistant to digestion, while the same molecules in metabolically active cells were fully digested. Northern blotting again confirmed the molecules to be 18S and 25S (Fig. 3b). Western blotting with an antibody specific for the phosphorylated S6 kinase (Fig. 3c) which gets phosphorylated directly by TOR, confirmed that TOR was inactivated in rapamycin treated cells. To show specifically that Pol I transcription of a full length rRNA was also downregulated we used an ITS2 specific probe. As can be seen by Northern blotting, no ITS2 could be detected after rapamycin exposure (Fig. 3d).

**Fig. 4:**
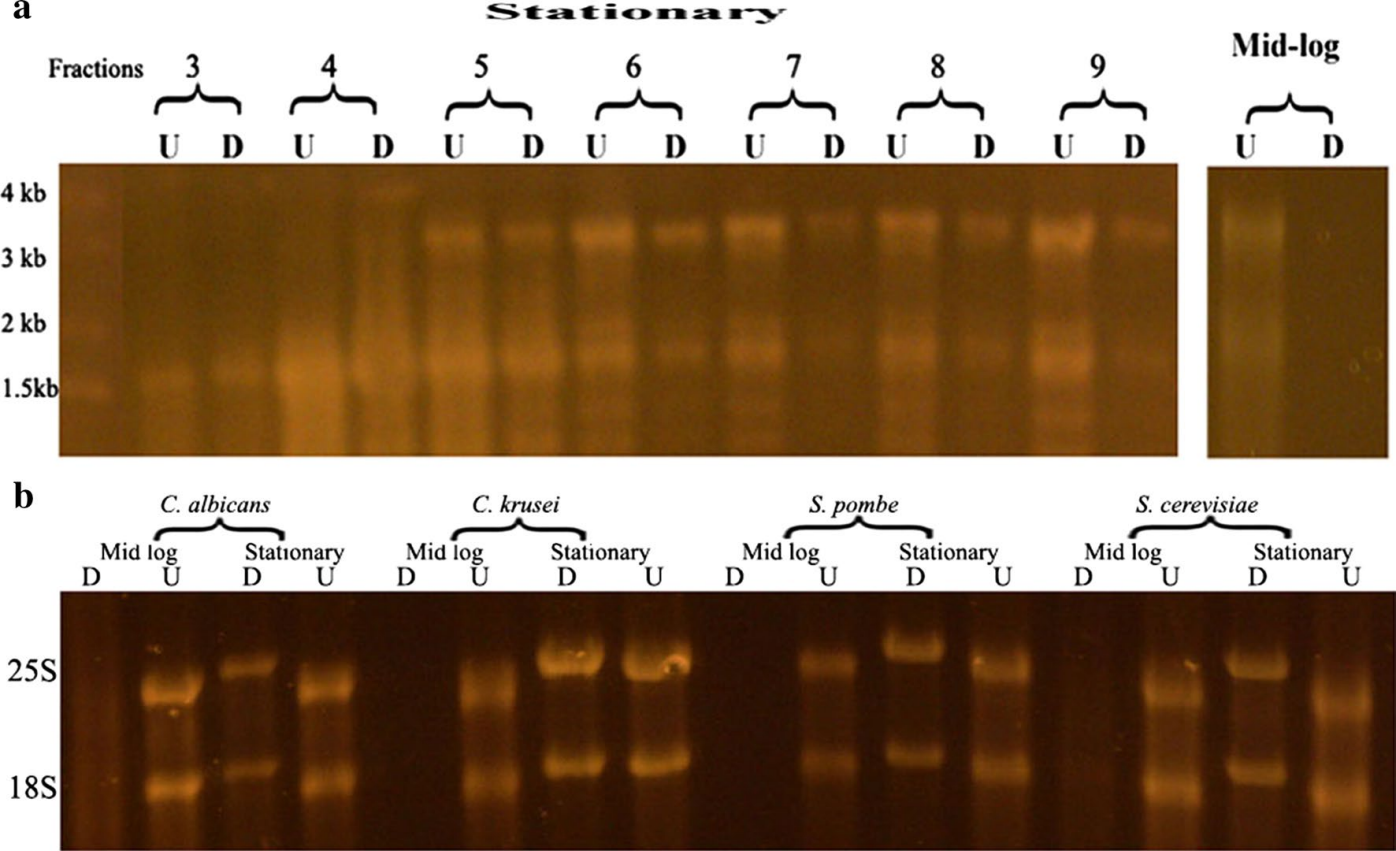
Incorporation of Terminator resistant 18S and 25S transcripts into ribosomes, and analysis of other yeasts for similar resistant rRNAs. **a** Agarose gel of RNA precipitated from isolated ribosomes from stationary phase cells. Numbers indicate fractions collected sequentially. Different elution fractions were combined prior to precipitation of RNA from isolated ribosomes from mid-log phase cells. Amount of Terminator digested RNA was the same as their undigested RNA pair. **b** SYBR gold stained gel containing RNA digested with Terminator (D) and undigested (U) isolated from mid log and stationary *Candida albican*s, *Candida krusei*, *Schizosaccharomyces pombe* and *Saccharomyces cerevisiae*

To answer the question whether these resistant molecules are utilized by *C. albicans* for ribosome production, we isolated ribosomes by centrifugation through a sucrose gradient. Following RNA purification and precipitation, we performed a Terminator treatment (Fig. 4a). Protected molecules for both 18S and 25S were seen in ribosomes from stationary organisms but not in ones isolated from mid-log phase organisms.

We isolated rRNA from mid-log and stationary organisms from three additional yeasts, namely, *Candida krusei*, *Schizosaccharomyces pombe*, and *Saccharomyces cerevisiae* and compared Terminator digested and undigested rRNAs. As can be seen on Fig. 4b, all three produced similar molecules in the stationary phase as did *C. albicans.*

## Discussion

Our data indicates that as *C. albicans* are encountering decreasing nutritional sources, they do something to 18S and 25S molecules to make them resistant to the Terminator exonuclease. We have repeated these experiments several times and the findings have been very consistent. The underlying mechanism for this change is unknown. Post-transcriptional modifications of rRNA such as pseudouridylation and methylation are well established [14] but they are unlikely to play a role here as these changes have been well mapped to rRNAs produced by mid-log phase yeast [15] where we find that Terminator is highly efficient in digesting them. What is most intriguing, is that both what is known about Terminator (http://www.epibio.com/enzymes/nucleases-glycosylases-dna-binding-proteins/rna-exonucleases/terminator-5-phosphate-dependent-exonuclease) and our additional digestion experiments, point toward the 5′-end of these molecules, where the enzymes encounters resistance. Most unexpected was that TAP treatment allowed Terminator to fully digest both 18S and 25S. This enzyme cuts specifically between phosphate groups which can result in a monophosphate 5′end exactly what is required by Terminator. If correct, this would indicate that there is an additional phosphate at the 5′-end of these molecules. Diphosphorylated mRNA molecules have recently been described in *Escherichia coli* (*E. coli*) [16]. These were initially triphosphorylated newly transcribed molecules, that were converted to a dephosphorylated state by a postulated enzyme that removed the γ-phosphate, which allowed for a more efficient RNA pyrophosphohydrolase (RppH) activity. In our case, 18S and 25S are molecules that are processed from a longer transcript and would need to have a phosphate group added to make them dephosphorylated. If true, this would be completely different from the process described for *E. coli*. Another possibility it raises is new transcription of these molecules. This would be most remarkable, as transcription and processing of rRNAs has been extensively studied and transcription of individual 18S and 25S has not been seen. Clearly, any new transcription and the identification of the polymerase doing it would require much more direct evidence.

Inhibition of TOR function by rapamycin resulting in similar 18S and 25S molecules reinforces our observations with nutritional depletion. It might indicate that their production is independent of TOR regulation or that their production is suppressed by TOR during adequate nutritional availability. We also show that the development of these resistant 18S and 25S molecules is not just an incidental process, since the cells incorporate them into their ribosomes. As organisms have their nutrition depleted they do enter the stationary phase which allows them longer survival [17, 18]. The stationary phase however is not a generalized decrease of all biochemical activity of the cell. For example, in *C. albicans*, a number of genes have been found to be expressed only in the stationary phase with some of them playing important roles in virulence [19]. Thus, it is not surprising that the organism will need to maintain some protein production capacity. By finding that these newly transcribed subunits are incorporated into the ribosome, makes it likely that in fact they are playing that role.

Finally, survey of other yeasts finds that they also produce the same molecules while approaching the stationary phase, indicating a conserved role for some biological processes for them.

## Conclusions

Terminator, a 5´-phosphate-dependent processive 5´→3´ exonuclease, has revealed 18S and 25S rRNA molecules that differ from the currently recognized ones processed from full length transcripts. They were found in yeasts approaching decreasing nutritional states and those with TOR inhibition with rapamycin. What is known about the enzyme and our data point to a change at the 5′ side of these molecules but the basis of this resistance is unknown. The finding of the same results in three different yeast species suggests a preserved role for these changes in the biology of these organisms.

## Methods

### Organisms

*Candida albican*s SC5314 (ATCC MYA 2876), *Candida krusei* (ATCC 14243), and *Saccharomyces Cerevisiae* AM109, (generous gift from Gera laboratory) were maintained in 50% glycerol in YPD broth (2% w/V tryptone, 1% w/v yeast extract, 2% w/v dextrose) at − 80 °C. Cells were activated in YPD broth at 30 °C and maintained on Sabouraud dextrose agar at 4 °C, passaged every 4–6 weeks up to 4–5 times. Yeasts were lifted from agar surface and grown in YPD broth for variable length of times at 30 °C. *Schizosaccharomyces Pombe* (genotype 972 h, strain FY7) was obtained from Forsburg laboratory. For some experiments 1 μg/ml of rapamycin (EMD Millipore) was added to fresh YPD. Yeast cell concentrations were established using a hemocytometer.

### RNA isolation

Cells were collected by centrifugation, washed with sterile phosphate buffered saline (PBS) and were put on ice pending total RNA extraction. Cells were disrupted with RNase-free zirconia beads and RNA was isolated using Ambion RiboPure RNA Purification kit for yeast (Ambion/ThermoFisher) according to the manufacturer’s instructions. RNA quantification was done using a Qubit 2.0 fluorometer.

### Terminator experiments

Total RNA was treated with Terminator (Epicentre) following the manufacturer’s protocol using Buffer B. The ratio of enzyme to substrate used was 1 U per 1 µg of RNA to ensure adequate cleavage.

### Northern blotting

The same amount of RNA was used for all conditions. RNA was separated on pre-fabricated formaldehyde agarose gels (Lonza) and stained with SYBR Gold Nucleic Acid Gel Stain (Life Technologies) for 30 min. Gel images were captured with a digital camera (Canon Vixia HFS30). RNA was transferred by electro-blotting (Thermo Scientific Owl Hep-1) to a positively charged nylon membrane (Life Technologies) in 0.5 × TBE (standard Tris/Borate/EDTA buffer). The RNA was cross-linked to the membrane using UV (Stratagene UV Crosslinker). Probes specific for 25S, 18S and 5S components of the ribosomal RNA were prepared by PCR using specific 18S, 25S and 5S primers. PCR products were cloned into TOPO^®^ pcr4 vector followed by transformation with TOP-10 chemically competent cells. Several colonies were screened for insert presence and sequenced (Laragen Inc). For probe preparation, bacteria were grown in Terrific Broth (Fisher) with ampicillin at 50 μg/ml, and plasmids were isolated with a QuickLyse Kit (Qiagen). Inserts were released with BamH I/EcoR I for 25S and 5S, and BamH I/NcoI for 18S, and were purified with a QIAquick Gel Extraction Kit (Qiagen). 50–100 ng of purified) inserts were biotinylated using the EZ-Link™ Psoralen-PEG3-Biotin (ThermoFisher Scientific). Membranes were pre-hybridized at 55 °C in North2South hybridization buffer (Pierce) for 30 min followed by overnight hybridization with denatured biotinylated probes at 5 ng/ml in the same buffer at 55 °C. Membranes were washed with low and high stringency buffers at room temperature and RNA was detected using Chemiluminescent Nucleic Acid Detection Kit (Pierce) according to the manufacturer’s protocol. Film was developed with the SRX-101A Konica film processor.

### AP/TAP/terminator analysis

RNA obtained from either stationary or mid-log organisms was treated with Alkaline phosphatase (Epicentre), Tobacco Acid pyrophosphatase (New England Bio) or 5′-Phosphate-Dependent Exonuclease (Terminator) (Epicentre) according to the manufacturer’s instructions. For experiments where sequential enzyme was required, RNA precipitation was performed immediately after first enzyme treatment using Acid-Phenol:Chloroform (Ambion) separation and ethanol precipitation. RNA quantity was established by Qubit^®^ measurement.

### Ribosome isolation and analysis

Stationary and mid-log C. *albicans* were grown in YPD at 30 °C. Ribosome isolation was done by following a previously described protocol [20]. Total protein concentration was measured using a Qubit^®^ fluorometer (Life Technologies). Extracts (0.2 ml) containing 2–4 mg of total protein were loaded onto the top of 7–47% sucrose gradient. Fractions of 1 ml were collected by pipetting from top of the column and placed in 1.7 ml tubes containing 100 µl 10% SDS. Each sample was digested with 11 µl proteinase K (20 mg/ml) for 30 min at 37 °C. Fractions of stationary RNA were precipitated individually while mid-log fractions were pooled prior to precipitation. RNA was measured using a Qubit^®^ fluorometer. RNA was digested with Teminator™ and electrophoresed through formaldehyde agarose gel along with undigested RNA as control.

## Abbreviations

TOR: target of rapamycin
ITS: intergenic transcribed spacer
ETS: external transcribed spacer
TAP: tobacco acid pyrophosphatase
AP: alkaline phosphatase
